# Development and reliability of metrics to characterize types and sources of stigma among men who have sex with men and female sex workers in Togo and Burkina Faso

**DOI:** 10.1186/s12879-019-3693-0

**Published:** 2019-03-05

**Authors:** Ashley L. Grosso, Sosthenes C. Ketende, Shauna Stahlman, Odette Ky-Zerbo, Henri Gautier Ouedraogo, Seni Kouanda, Cesaire Samadoulougou, Marcel Lougue, Jules Tchalla, Simplice Anato, Sodji Dometo, Felicity D. Nadedjo, Vincent Pitche, Stefan D. Baral

**Affiliations:** 1 Key Populations Program, Center for Public Health and Human Rights, Department of Epidemiology, Johns Hopkins Bloomberg School of Public Health, 615 North Wolfe Street, 5th Floor, Baltimore, MD USA; 2 Research and Evaluation Unit, Public Health Solutions, 40 Worth Street, 5th Floor, New York, NY USA; 3 Programme d’Appui au Monde Associatif et Communautaire (PAMAC), 11 BP 1023, Avenue du Pr Joseph Ki-Zerbo, Ouagadougou, Burkina Faso; 4 Institut de Recherche en Sciences de la Santé (IRSS), 03 BP 7192, Ouagadougou, 03 Burkina Faso; 5 Lomé, Togo; 6 Arc-en-ciel, BP 805000, Lomé, Togo; 7 FAMME, BP 12.321 Ville, Lomé, Togo; 8 Conseil National de Lutte contre le SIDA et les IST, 01 BP 2237, Lomé, 01 Togo

**Keywords:** Burkina Faso, Social stigma, Sex workers, HIV, MSM, Sexual minorities, Togo

## Abstract

**Background:**

Stigma is a multifaceted concept that potentiates Human Immunodeficiency Virus and sexually transmitted infection acquisition and transmission risks among key populations, including men who have sex with men (MSM) and female sex workers (FSW). Despite extensive stigma literature, limited research has characterized the types and sources of stigma reported by key populations in Sub-Saharan Africa.

**Methods:**

This study leveraged data collected from 1356 MSM and 1383 FSW in Togo and Burkina Faso, recruited via respondent-driven sampling. Participants completed a survey instrument including stigma items developed through systematic reviews and synthesis of existing metrics. Using exploratory factor analysis with promax oblique rotation, 16 items were retained in a stigma metric for MSM and 20 in an FSW stigma metric. To assess the measures’ convergent validity, their correlations with expected variables were examined through bivariate logistic regression models.

**Results:**

One factor, experienced stigma, included actions that were carried out by multiple types of perpetrators and included being arrested, verbally harassed, blackmailed, physically abused, tortured, or forced to have sex. Other factors were differentiated by source of stigma including healthcare workers, family and friends, or police. Specifically, stigma from healthcare workers loaded on two factors: experienced healthcare stigma included being denied care, not treated well, or gossiped about by healthcare workers and anticipated healthcare stigma included fear of or avoiding seeking healthcare. Stigma from family and friends included feeling excluded from family gatherings, gossiped about by family, or rejected by friends. Stigma from police included being refused police protection and items related to police confiscation of condoms. The Cronbach’s alpha ranged from 0.71–0.82. Median stigma scores, created for each participant by summing the number of affirmative responses to each stigma item, among MSM were highest in Ouagadougou and among FSW were highest in both Ouagadougou and Bobo-Dioulasso. Validation analyses demonstrated higher stigma was generally significantly associated with suicidal ideation, disclosure of involvement in sex work or same-sex practices, and involvement in organizations for MSM or FSW.

**Conclusions:**

Taken together, these data suggest promising reliability and validity of metrics for measuring stigma affecting MSM and FSW in multiple urban centers across West Africa.

## Background

Stigma has been defined as processes of labeling, stereotyping, separation, status loss, and discrimination of individuals or groups [1]. Some theories include both psychological and social dimensions of stigma but exclude discrimination from the definition [2]. Structural conceptualizations of stigma recognize that macro-level forces also compound marginalization by placing constraints on affected groups themselves as well as influencing social interactions with affected populations [3]. Several stigma types have been characterized and studied [4, 5]. Perceived (also called felt [6], or felt-normative [7]) stigma refers to the impression or belief that individuals or societies treat people differently due to stigmatized characteristics. Enacted or experienced stigma [8, 9] represents the explicit experience of poor treatment on the basis of specific characteristics. Internalized or self-stigma is the acceptance of one’s perceived lesser status within society and manifests in low self-esteem, self-isolation, and social withdrawal [10, 11]. Finally, anticipated stigma [12] is the fear or expectation of discrimination. This is distinct from stigma toward oneself and involves worry by the stigmatized individual, which may or may not be based on others’ actual actions. Secondary [13] or courtesy stigma [5] occurs when people associated with the person being stigmatized are also exposed to stigma. Finally, intersecting or compounded stigmas is the experience or perception of multiple forms of stigmas, such as that stigma often experienced related to identifying as gay or openly living with Human Immunodeficiency Virus (HIV) [4].

After years of consistent data, there is consensus that social and structural factors, including stigma, potentiate HIV risks by limiting the provision and uptake of evidence-based HIV prevention, treatment, and care services [14]. Stigma affecting populations with disproportionate HIV burden has consistently been associated with negative health outcomes throughout the world. In Uganda, experiencing homophobic abuse was positively associated with HIV infection among men who have sex with men (MSM) [15]. Occupational stigma among female sex workers (FSW) in Canada was correlated with experiencing barriers to healthcare access [16]. In The Gambia, internal and experienced stigma toward people living with HIV were associated with poorer self-reported health [17]. At the public policy level, stigma may be related to criminalization of key populations’ practices and lower levels of funding of specific health services for key populations because they are deemed unworthy of assistance [18–20].

Reliable and valid stigma metrics are needed to assess correlates of stigma and changes in stigma over time [21] in order to evaluate stigma mitigation interventions [22] and assess dose-response associations of stigma with health outcomes. However, there are several gaps in the research literature on measuring stigma specifically among key populations. This is especially the case across Sub-Saharan Africa (SSA) [23–28]. Previous studies have often focused on investigating stigma from others’ perspectives rather than from MSM and FSW themselves [29, 30]. Additionally, many existing studies using or evaluating metrics have focused on assessing HIV-related stigma among key populations [31], though fewer in these contexts have focused on stigma related to selling sex or same-sex sexual practices, identities, relationships or communities [32–37]. Existing studies have often been completed in high or upper middle income countries [35, 38–41]. In a recent systematic review on stigma measures, only three articles included both MSM and FSW participants [42]. Of seven articles measuring stigma reported by FSW study participants, five were about perceived stigma, one was about internalized stigma, and one was about experienced stigma. These surveys did not ask the source of this stigma (e.g., family, friends, healthcare workers, or police). None of these studies took place in Sub-Saharan Africa. While more measures of stigma toward MSM were identified, only four were from Sub-Saharan Africa (all from South Africa).While the HIV epidemics, especially in Southern and Eastern Africa, are more broadly generalized, key populations have consistently been shown to have higher burdens of HIV than other reproductive aged men [43, 44] and women [45].

In West and Central Africa, the most populous region of SSA, HIV is more concentrated among key populations including MSM and FSW compared to what has historically been observed in Southern and Eastern Africa [43]. Across the countries of West Africa, enforcement of punitive laws related to MSM and FSW varies significantly. In Burkina Faso, same-sex practices are not mentioned in the law. Selling sex is tolerated and not prohibited; soliciting and facilitating sex work are criminalized [46, 47]. However, stigma related to HIV [48, 49], sex work [50] and same-sex practices [51] is common. Socially and in the media there are strong expressions against same-sex sexual practices and relationships. For example, 95% of participants from Burkina Faso in the Afrobarometer said they would dislike living next to gay or lesbian neighbors [52]. A bill was introduced (and later rejected) to criminalize same-sex practices. There have been public marches against same-sex sexual practices and relationships [53]. Sex work and same-sex practices are both criminalized in Togo. Specifically, same-sex practices may be punished with fines between 100,000–500,000 XOF ($200–1000) and 1 to 3 years imprisonment [54]. Selling sex in specific sex work venues is prohibited, with a maximum fine of 1,000,000 XOF ($2000); however compensated or transactional sex is not specifically prohibited [55].

In these settings, it remains vital to characterize appropriate tools to measure stigma and inform evaluating the impact of stigma mitigation interventions. Consequently, this study aims to characterize the reliability and convergent validity of stigma metrics for MSM and FSW in two West African countries.

## Methods

### Settings of the study/study sites

Data were collected from January–August 2013 using respondent-driven sampling of 1356 MSM and 1383 FSW in Ouagadougou, and Bobo-Dioulasso, Burkina Faso and Lomé, and Kara, Togo, West Africa.

### Type of study

Details of the study procedures have been previously reported [56–66]. Briefly, the original purposes of these studies were to develop population-based estimates of HIV prevalence and determinants of those living with HIV at the individual, network, community and policy levels.

### Participants

Inclusion criteria for FSW included being assigned the female gender at birth and reporting most of their income came from selling sex in the past year. Eligible MSM participants were assigned the male gender at birth and had anal sex with a man in the past year. All participants were 18 years old or older and lived in the respective city for at least the past 3 months. Respondent-driven sampling coupons were required for all participants (except for seeds). Participants in Burkina Faso provided written informed consent in French, Mooré, or Dioula. In Togo, participants provided oral informed consent in French, Ewe or Kabiye.

### Data collection

Trained interviewers administered structured questionnaires including modules on demographics, stigma, HIV-related knowledge and behaviors, mental health, social capital, access to services, and reproductive health.

### Stigma measurement

Questions related to stigma were initially informed through literature reviews, consultations with key stakeholders including social scientists and nongovernmental organizations, and have been adapted over a decade of studies with key populations across SSA [9, 67–82]. Listwise deletion was used to handle missing data, including “don’t know” and “refusal” responses.

### Analyses

The analytic approach chosen was exploratory factor analysis [83] given the lack of prior studies with factor analysis of these items. Despite the rich body of prior stigma scale development, this inductive method was used because most of stigma measurement research with key populations [42] has been conducted with Western, Educated, Industrialized, Rich, or Democratic (WEIRD) [84] samples or societies. Other psychometric instruments, such as personality measures, have been found to have different factor structures outside of these WEIRD settings [85]. A recent study comparing reports of stigma from surveys administered to MSM in the United States and Western and Southern Africa found differences between settings in prevalence of family exclusion, poor health care treatment, and blackmail but did not use factor analysis to compare the structure of stigma measures [86].

Principal factor analysis (or common factor analysis) was used because the goal was not data reduction but rather to describe the underlying latent stigma variables [87]. Promax oblique rotation [88] was used because the factors were hypothesized to be correlated [89]. Data from each city and population were analyzed separately using Stata 13.1 (College Station, Texas) because the respondent-driven sampling networks were different by city and because of the potential value of metrics that can be used with multiple populations in multiple geographic areas. Results using combined data were similar to those reported below. The sample size in each dataset was sufficient to allow for greater than the recommended ten participants per variable [87]. The Kaiser-Meyer-Olkin [90] measure of sampling adequacy was used with the Bartlett’s test of sphericity [91] to assess the correlation matrices’ suitability for factor analysis. The factors retained were based on Kaiser’s criterion [92], eigenvalues over one, scree plots, Horn’s test [93], and interpretability. Items considered for the final metrics had factor loadings greater than 0.3 [94] in at least one city for each population. This was done to allow for comparisons across groups. Questions were excluded if they loaded on more than one factor [95] or variability in the distributions of responses was under 10% [96]. Questions were also excluded if greater than 20% of participants had missing data, as this may indicate they were misunderstood or particularly sensitive [97, 98]. These questions, shown in Table 1 with the rationales for excluding them, were about discrimination in employment and education, being forced to test for HIV, being scared to walk in public places, difficulties accessing healthcare for FSW, and hearing discriminatory remarks about same-sex sexual practices and relationships for MSM. Stigma scores were categorized by parceling [99], adding the remaining questions such that participants who answered “yes” to one included question were assigned a score of 1, participants who answered “yes” to two questions were assigned a score of 2, and so on. This technique was chosen because of its simplicity and practical use for community organization staff who may not have the skills or software to compute factor scores [100].

**Table 1:** Items included and excluded from FSW^a^ and MSM^b^ stigma metrics in Burkina Faso and Togo

Variable name	Question text
Police harassed	Have police ever harassed or intimidated you for being a sex worker?
Arrested	Were you ever arrested on charges **related to sex work***/ of homosexuality [or other related charge]*?
Verbally harassed	Have you ever been verbally harassed and felt it was because you **sell sex**/ *have sex with men*?
Blackmailed	Have you ever been blackmailed by someone because you **sell sex**/ *have sex with men*?
Physically abused	Have you ever been physically aggressed (pushed, shoved; slapped; hit; kicked; choked; or otherwise physically hurt)? Do you believe any of these experience(s) of physical violence was/were related to the fact that you **sell sex**/ *have sex with men*?
Tortured	Have you ever been tortured by someone? If yes, do you believe this was because you **sell sex**/ *have sex with men*?
Forced sex	Have you ever been forced to have sex when you did not want to? (By forced, I mean physically forced, coerced to have sex, or penetrated with an object, when you did not want to). Do you believe any of these experiences of sexual violence were related to the fact that you **sell sex**/ *have sex with men*?
Denied care	Have you ever been denied health services (or someone kept you from receiving health services) because you **sell sex**/ *have sex with men*?
Not treated well	Have you ever felt that you were not treated well in a health center because **you sell sex**/ *someone knew that you have sex with men*?
Health workers gossiped	Have you ever heard healthcare providers gossiping about you because you **sell sex**/ *have sex with men*?
Difficulties	Have you ever had difficulties in accessing healthcare services because you have sex with men?
Afraid to seek care	Have you ever felt afraid to go to healthcare services because you worry someone may learn you **sell sex**/ *have sex with men*?
Avoided care	Have you ever avoided going to healthcare services because you worry someone may learn you **sell sex**/ *have sex with men*?
Family excluded	Have you ever felt excluded from family gatherings because you **sell sex**/ *have sex with men*?
Family gossiped	Have you ever felt that family members have made discriminatory remarks or gossiped about you because you **sell sex**/ *have sex with men*?
Friends rejected	Have you ever felt rejected by your friends because you **sell sex**/ *have sex with men*?
Police refused	Have you ever felt that the police refused to protect you because you **sell sex**/ *have sex with men*?
Avoided carrying condoms	Have you ever avoided carrying condoms because you were afraid that they might get you in trouble with the police?
Police confiscated	Has a police officer ever taken condoms away from you, thrown them on the ground or in the garbage?
Witnessed confiscation	Have you ever witnessed (i.e. seen) police confiscating or destroying condoms held by a sex worker or outreach worker?
Heard about confiscation	Have you ever heard about incidents when police confiscated or destroyed condoms held by other sex workers or by outreach workers?
Question	Reason for excluding
Female sex workers	
Have you ever lost employment or been dismissed from a job (other than sex work) because you sell sex?	Many participants answered “not applicable”; did not load strongly on any factor
Have you ever been denied educational opportunities, like access to school, because you sell sex?	Many participants answered “not applicable”; did not load strongly on any factor
Have you ever had difficulties in accessing healthcare services because you sell sex?	High uniqueness; did not load on any factor in multiple datasets
Have you ever been tested for HIV when you did not want to or did not give permission? If yes, were you forced or pressured to test for HIV because you sell sex?	Very rare (9 people in all cities combined)
Have you ever refused to take condoms from an outreach worker because you were afraid they might get you in trouble with the police?	Very rare (17 total in all cities combined)
Have you ever felt scared to walk around in public places because you sell sex?	Did not load on any factor; may be measuring something other than stigma
Men who have sex with men	
Have you ever lost employment or been dismissed from a job because you have sex with men?	Loaded on a different factor in every city
Have you ever been denied educational or training opportunities, like access to school, because you have sex with men?	Rare (10 people or less per city); did not load on any factors; lowered the Cronbach’s alpha of the metric
Has anyone ever said discriminatory things about homosexuality in your presence without knowing you have sex with other men?	Did not load on any factors; lowered the Cronbach’s alpha
Have you ever been tested for HIV when you did not want to or did not give permission? If yes, were you forced or pressured to test for HIV because you have sex with men?	Very rare (only 8 people in all cities combined)

Bold = wording in the female sex worker questionnaire

Italics = wording in the men who have sex with men questionnaire

^a^female sex worker

^b^men who have sex with men

While some other stigma metrics are comprised of Likert items [101], which may increase internal consistency and capture more fine-grained differences in frequency of exposure to stigma, the metrics in the present study are based on dichotomous items. This approach enables assessment of the prevalence and correlates of lifetime experiences of stigma, can be faster to administer (which is important in the context of a long questionnaire that includes multiple topics in addition to stigma), and may not result in reduced validity or test-retest reliability [102]. The present study’s measures’ reliability was assessed using Cronbach’s alpha [103] and the Kuder-Richardson coefficient [104]. The overall metrics’ reliability was acceptable, with Cronbach’s alpha coefficients ranging from 0.71 among MSM in Ouagadougou to 0.82 among FSW in Ouagadougou. The Kuder-Richardson coefficients were the same as the Cronbach’s alpha except in Lomé, Togo where for the FSW metric the alpha was 0.73 and the Kuder-Richardson coefficient was 0.70.

To assess the measures’ convergent validity, their correlations with expected HIV-related determinants were examined. This was done to evaluate whether the stigma metrics are useful not only for assessing levels of stigma but also within models with other HIV-related risk determinants. The literature suggests stigma would be associated with increased purposeful or inadvertent disclosure represented by family members or health workers knowing about the participant’s sex work or same-sex practices [75, 105]. This study also tested whether higher stigma is related to having suicidal ideation [32, 106]. Among participants recruited into the study as MSM, it was further hypothesized that those who identified as men would have lower stigma scores than those who identified as transgender, female or intersex [107]. It was expected that higher stigma would be inversely associated with HIV testing and condom use [26, 108]. Stigma scores were used as continuous independent variables in all models. Separate bivariate logistic regression models were used to estimate the relationship between stigma and dichotomous dependent variables (disclosure to family or health workers of selling sex for FSW and having sex with men or being attracted to men for MSM, suicidal ideation, participating in MSM/FSW/HIV organizations, and gender identity). Results were considered statistically significant at *p* < 0.05 using two-sided tests.

The Johns Hopkins School of Public Health Institutional Review Board, Burkina Faso Ethics Committee for Health Research, and Togo National Ethics Committee approved the study.

## Results

The median age of FSW participants ranged from 23 years old in Kara and Ouagadougou to 30 in Bobo-Dioulasso (see Table 2). Fewer than half of FSW in Burkina Faso and over 70% in Togo completed primary school or higher. The proportion employed outside of selling sex ranged from 7.1% in Bobo-Dioulasso to 53.4% in Lomé. Under 15% were married or cohabitating. Over half had at least one biological child. One third said a family member knew about their involvement in sex work. One fifth voluntarily disclosed. Over one quarter disclosed to a health worker, and 10% reported a health worker found out some other way. Over one fifth ever had suicidal ideation. Less than half had condomless vaginal sex in the past 12 months. Most had tested for HIV more than once ever.

**Table 2:** Selected characteristics of female sex workers in Burkina Faso and Togo

City	Ouagadougou	Bobo-Dioulasso	Lomé	Kara
Median age	23	30	28	23
Completed primary school or higher	46.1% (159/345)	27.7% (97/350)	70.9% (251/354)	86.1% (284/330)
Employed (other than sex work)	33.1% (115/347)	7.1% (25/350)	53.4% (189/354)	47.0% (155/330)
Married or cohabitating	9.5% (33/349)	12.3% (43/350)	7.3% (26/354)	5.8% (19/330)
Has at least one biological child	69.3% (242/349)	83.4% (292/350)	78.5% (278/354)	51.2% (169/330)
Told family about sex work	16.3% (57/349)	22.3% (78/350)	19.5% (69/354)	21.5% (71/330)
Family found out about sex work	31.0% (102/329)	32.9% (109/331)	19.4% (65/335)	48.8% (157/322)
Told health worker about sex work	16.1% (55/342)	22.8% (79/347)	45.0% (159/353)	26.1% (86/330)
Health worker found out about sex work	12.9% (44/342)	6.3% (22/347)	15.0% (53/353)	6.1% (20/330)
Ever had suicidal thoughts	20.1% (70/349)	22.9% (80/350)	21.2% (75/354)	17.9% (59/330)
Participated in female sex worker organization	13.9% (48/346)	24.6% (86/350)	24.4% (86/353)	3.3% (11/330)
Had condomless vaginal sex in the past 12 months	37.9% (130/343)	43.3% (151/349)	25.5% (87/341)	46.2% (151/327)
Ever tested for HIV more than once	60.2% (209/347)	68.3% (239/350)	58.6% (205/350)	56.1% (185/330)

Across all the cities, the median age of participants in the MSM study was under 25 years old (see Table 3). Over 90% completed primary school or higher. Most (> 50%) were unemployed (including students). Under 10% were married or cohabitating with a woman or had a biological child. The majority reported their sexual orientation as gay. One fifth said a family member knew about their sexual practices. One quarter disclosed to them. Under 5% reported a health worker found out about their sexual practices. One fifth voluntarily disclosed. Over 10% reported suicidal ideation. Over one quarter identified their gender as transgender, female or intersex. Most had ever had condomless anal sex. Over half had tested for HIV more than once ever.

**Table 3:** Selected characteristics of men who have sex with men (MSM) in Burkina Faso and Togo

City	Ouagadougou	Bobo-Dioulasso	Lomé	Kara
Median age	21	22	22	24
Completed primary school or higher	93.0% (319/343)	90.3% (298/330)	99.2% (351/354)	99.4% (327/329)
Employed	19.5% (67/343)	26.4% (87/330)	43.5% (154/354)	23.1% (76/329)
Married or cohabitating with a woman	5.0% (17/340)	3.0% (10/329)	8.5% (30/354)	3.0% (10/329)
Has at least one biological child	7.6% (26/343)	7.9% (26/330)	5.4% (19/353)	1.8% (6/329)
Sexual orientation:				
Gay or homosexual	51.3% (176/343)	55.8% (184/330)	61.0% (216/354)	68.7% (226/329)
Bisexual	44.0% (151/343)	39.4% (130/330)	35.0% (124/354)	31.3% (103/329)
Heterosexual or straight	2.0% (7/343)	3.9% (13/330)	0.8% (3/354)	0.0% (0/329)
Transvestite/transgender	2.6% (9/343)	0.9% (3/330)	3.1% (11/354)	0.0% (0/329)
Told family about same-sex practices	26.0% (89/343)	20.3% (67/330)	24.0% (85/354)	29.8% (98/329)
Family found out about same-sex practices	18.1% (58/321)	19.3% (62/321)	18.0% (62/344)	29.1% (93/320)
Told health worker about same-sex practices	19.9% (66/332)	20.0% (61/305)	36.3% (128/353)	8.8% (29/329)
Health worker found out about same-sex practices	3.3% (11/332)	2.3% (7/305)	10.5% (37/353)	1.5% (5/329)
Ever had suicidal thoughts	14.9% (51/343)	17.0% (56/329)	13.9% (49/353)	6.1% (20/329)
Participated in MSM organization	17.0% (58/342)	8.0% (26/327)	40.7% (144/354)	1.22% (4/329)
Identifies as male	70.8% (242/342)	61.2% (202/330)	72.0% (255/354)	91.5% (301/329)
Ever had condomless anal sex	50.4% (173/343)	67.9% (222/327)	43.8% (155/354)	53.8% (177/329)
Ever tested for HIV more than once	51.6% (177/343)	55.2% (181/328)	55.9% (195/349)	47.4% (156/329)

The Bartlett’s test of sphericity was significant (*p* < 0.05), indicating the data were suitable for factor analysis. The Kaiser-Meyer-Olkin index was greater than the minimum acceptable value of 0.5 in all datasets. A five-factor solution was identified for the FSW metric (20 questions total) and a four-factor solution for the MSM metric (16 questions total). Two to seven variables loaded on each factor (Tables 4 and 5). The factors were each significantly correlated with one another (*p* < 0.001).

**Table 4:** Stigma item frequencies in the female sex worker stigma metric in Burkina Faso and Togo

City	Ouagadougou (*n* = 349)	Bobo-Dioulasso (*n* = 350)	Lomé (*n* = 354)	Kara (*n* = 330)
Factor 1: Experienced stigma				
1. Police harassed	28.9% (101/349)	48.4% (169/349)	29.7% (105/354)	22.4% (74/330)
2. Arrested	32.6% (113/347)	44.8% (155/346)	25.1% (89/354)	13.3% (44/330)
3. Verbally harassed	63.6% (222/349)	55.4% (194/350)	35.9% (127/354)	37.3% (123/330)
4. Blackmailed	19.9% (69/347)	42.0% (147/350)	20.6% (73/354)	36.2% (119/329)
5. Physically abused	49.9% (163/327)	44.2% (153/346)	22.0% (78/354)	19.4% (64/330)
6. Tortured	13.0% (44/338)	17.6% (61/346)	11.6% (41/354)	4.9% (16/330)
7. Forced sex	27.5% (95/346)	29.1% (102/350)	11.0% (39/354)	16.1% (53/329)
Factor 2: Experienced healthcare stigma				
1. Denied care	1.2% (4/349)	0.9% (3/350)	0.6% (2/354)	0.0% (0/330)
2. Not treated well	2.0% (7/349)	1.7% (6/349)	1.7% (6/354)	2.7% (9/330)
3. Health workers gossiped	6.6% (23/349)	2.3% (8/350)	1.4% (5/354)	5.8% (19/330)
Factor 3: Anticipated healthcare stigma				
1. Afraid to seek care	21.0% (73/348)	14.9% (52/349)	5.7% (20/353)	10.0% (33/330)
2. Avoided care	15.5% (54/349)	14.9% (52/349)	4.5% (16/354)	9.7% (32/330)
Factor 4: Stigma from family and friends				
1. Family excluded	23.0% (80/348)	16.1% (56/349)	4.2% (15/354)	18.8% (62/330)
2. Family gossiped	33.8% (117/346)	30.4% (105/345)	8.5% (30/354)	36.8% (121/329)
3. Friends rejected	16.8% (58/345)	19.4% (68/350)	4.5% (16/354)	20.3% (67/330)
Factor 5: Stigma from police				
1. Police refused	18.4% (64/347)	16.4% (57/348)	8.5% (30/353)	2.4% (8/330)
2. Avoided carrying condoms	12.3% (43/349)	6.3% (22/349)	3.4% (12/354)	0.9% (3/330)
3. Police confiscated	5.2% (18/348)	6.9% (24/346)	2.5% (9/354)	0.3% (1/330)
4. Witnessed confiscation	7.2% (25/347)	5.4% (19/350)	7.1% (25/354)	3.9% (13/330)
5. Heard about confiscation	13.3% (46/347)	17.7% (62/350)	36.5% (129/353)	7.3% (24/330)

**Table 5:** Stigma Item Frequencies in the MSM^a^ Stigma Metric in Burkina Faso and Togo

City	Ouagadougou (*n* = 343)	Bobo-Dioulasso (*n* = 330)	Lomé (*n* = 354)	Kara (*n* = 329)
Factor 1: Experienced stigma				
1. Police refused	5.2% (18/342)	3.3% (11/330)	3.7% (13/354)	0.3% (1/329)
2. Arrested	1.7% (6/343)	1.2% (4/329)	2.8% (10/354)	0.3% (1/329)
3. Verbally harassed	34.7% (119/343)	44.7% (147/329)	18.9% (67/354)	18.2% (60/329)
4. Blackmailed	24.8% (85/343)	14.9% (49/329)	16.1% (57/354)	21.9% (72/329)
5. Physically abused	9.6% (33/343)	15.3% (50/327)	4.2% (15/354)	4.0% (13/329)
6. Tortured	2.9% (10/343)	2.8% (9/326)	3.1% (11/354)	3.0% (10/329)
7. Forced sex	10.5% (36/342)	10.7% (35/328)	3.7% (13/354)	4.3% (14/329)
Factor 2: Experienced healthcare stigma				
1. Denied care	1.5% (5/342)	0.9% (3/330)	1.1% (4/354)	0.0% (0/329)
2. Not treated well	4.4% (15/343)	3.3% (11/330)	2.0% (7/354)	0.6% (2/329)
3. Health workers gossiped	12.0% (41/343)	2.7% (9/330)	3.4% (12/354)	6.7% (22/329)
4. Difficulties	7.3% (25/343)	1.8% (6/330)	17.0% (60/354)	7.3% (24/329)
Factor 3: Anticipated healthcare stigma				
1. Afraid to seek care	40.8% (140/343)	23.6% (78/330)	8.5% (30/354)	11.3% (37/329)
2. Avoided care	36.4% (125/343)	20.0% (66/330)	7.1% (25/354)	9.1% (30/329)
Factor 4: Stigma from family and friends				
1. Family excluded	12.2% (42/343)	8.2% (27/330)	6.5% (23/354)	12.2% (40/329)
2. Family gossiped	37.3% (128/343)	26.4% (87/329)	15.3% (54/354)	19.5% (64/329)
3. Friends rejected	35.9% (123/343)	26.1% (86/330)	8.8% (31/354)	16.4% (54/329)

^a^men who have sex with men

One factor, experienced stigma, included actions that were carried out by multiple types of perpetrators. Other factors were differentiated by source of stigma (healthcare workers, family and friends, or police). Stigma from healthcare workers loaded on two factors. The factors common to both populations were: stigma from family and friends; experienced stigma; experienced healthcare stigma; and anticipated healthcare stigma. The fifth factor in the FSW metric was stigma from police (Tables 6 and 7).

**Table 6:** MSM^a^ stigma metric factor structure and loadings in Burkina Faso and Togo

City	Ouagadougou	Bobo-Dioulasso	Lomé	Kara
	Alpha = 0.59 Variance = 1.58 Proportion = 37%	Alpha = 0.48 Variance = 1.36 Proportion = 24%	Alpha = 0.64 Variance = 2.29 Proportion = 39%	Alpha = 0.63 Variance = 2.14 Proportion = 36%
Factor 1: Experienced stigma				
1. Police refused	0.471	0.530	0.123	0.995
2. Arrested	0.588	0.572	0.291	0.995
3. Verbally harassed	0.121	0.105	0.605	−0.004
4. Blackmailed	0.175	0.124	0.481	−0.006
5. Physically abused	0.206	0.194	0.096	−0.080
6. Tortured	0.562	−0.126	0.281	0.260
7. Forced sex	0.410	0.074	0.646	−0.076
	Alpha = 0.39 Variance = 1.53 Proportion = 36%	Alpha = 0.78 Variance = 2.69 Proportion = 46%	Alpha = 0.33 Variance = 1.24 Proportion = 21%	Alpha = 0.18 Variance = 1.87 Proportion = 27%
Factor 2: Experienced healthcare stigma				
1. Denied care	0.199	0.808	0.488	N/A
2. Not treated well	0.564	0.694	0.464	0.178
3. Health workers gossiped	0.370	0.697	0.474	0.196
4. Difficulties	0.249	0.700	0.239	0.051
	Alpha = 0.87 Variance = 1.51 Proportion = 35%	Alpha = 0.92 Variance = 2.22 Proportion = 38%	Alpha = 0.88 Variance = 2.12 Proportion = 36%	Alpha = 0.88 Variance = 1.75 Proportion = 25%
Factor 3: Anticipated healthcare stigma				
1. Afraid to seek care	0.833	0.913	0.832	0.850
2. Avoided care	0.837	0.907	0.794	0.859
	Alpha = 0.55 Variance = 2.01 Proportion = 47%	Alpha = 0.60 Variance = 1.70 Proportion = 29%	Alpha = 0.77 Variance = 2.89 Proportion = 50%	Alpha = 0.76 Variance = 2.52 Proportion = 36%
Factor 4: Stigma from family and friends				
1. Family excluded	0.352	0.580	0.737	0.748
2. Family gossiped	0.648	0.619	0.748	0.773
3. Friends rejected	0.521	0.509	0.720	0.589
Total	Alpha = 0.71	Alpha = 0.71	Alpha = 0.77	Alpha = 0.75
	KR^b^=0.71	KR = 0.71	KR = 0.77	KR = 0.75
Mean (SD)	2.753 (2.407)	2.053 (2.151)	1.220 (1.971)	1.350 (1.975)
Median	2	1	0	0

^a^men who have sex with men

^b^Kuder-Richardson coefficient

**Table 7:** Female sex worker stigma metric factor structure and loadings in Burkina Faso and Togo

City	Ouagadougou	Bobo-Dioulasso	Lomé	Kara
	Alpha = 0.72 Variance = 2.97 Proportion = 41%	Alpha = 0.69 Variance = 2.82 Proportion = 36%	Alpha = 0.75 Variance = 2.60 Proportion = 38%	Alpha = 0.76 Variance = 2.80 Proportion = 39%
Factor 1: Experienced stigma				
1. Police harassed	0.485	0.510	0.531	0.612
2. Arrested	0.574	0.600	0.557	0.500
3. Verbally harassed	0.485	0.280	0.638	0.598
4. Blackmailed	0.411	0.403	0.506	0.671
5. Physically abused	0.695	0.371	0.671	0.612
6. Tortured	0.216	0.127	0.563	0.409
7. Forced sex	0.377	0.399	0.421	0.542
	Alpha = 0.37 Variance = 1.18 Proportion = 16%	Alpha = 0.72 Variance = 1.95 Proportion = 25%	Alpha = 0.56 Variance = 1.56 Proportion = 23%	Alpha = 0.31 Variance = 0.97 Proportion = 14%
Factor 2: Experienced healthcare stigma				
1. Denied care	0.558	0.849	0.675	N/A
2. Not treated well	0.200	0.814	0.728	0.285
3. Health workers gossiped	0.365	0.409	0.308	0.463
	Alpha = 0.89 Variance = 2.53 Proportion = 35%	Alpha = 0.85 Variance = 2.62 Proportion = 33%	Alpha = 0.74 Variance = 1.67 Proportion = 25%	Alpha = 0.92 Variance = 2.06 Proportion = 29%
Factor 3: Anticipated healthcare stigma				
1. Afraid to seek care	0.889	0.856	0.648	0.894
2. Avoided care	0.883	0.858	0.764	0.914
	Alpha = 0.76 Variance = 2.75 Proportion = 38%	Alpha = 0.73 Variance = 3.31 Proportion = 42%	Alpha = 0.59 Variance = 1.17 Proportion = 17%	Alpha = 0.68 Variance = 1.76 Proportion = 25%
Factor 4: Stigma from family and friends				
1. Family excluded	0.816	0.842	0.590	0.625
2. Family gossiped	0.815	0.802	0.592	0.700
3. Friends rejected	0.423	0.436	0.163	0.449
	Alpha = 0.67 Variance = 2.79 Proportion = 39%	Alpha = 0.69 Variance = 3.39 Proportion = 43%	Alpha = 0.32 Variance = 1.28 Proportion = 19%	Alpha = 0.48 Variance = 1.54 Proportion = 22%
Factor 5: Stigma from police				
1. Police refused	0.327	0.284	0.113	−0.070
2. Avoided carrying condoms	0.430	0.551	0.088	0.034
3. Police confiscated	0.583	0.614	0.649	0.245
4. Witnessed confiscation	0.672	0.818	0.637	0.788
5. Heard about confiscation	0.712	0.678	0.369	0.816
Total	Alpha = 0.82	Alpha = 0.82	Alpha = 0.73	Alpha = 0.77
	KR^a^=0.82	KR = 0.82	KR = 0.70	KR = 0.77
Mean (SD)	4.313 (3.651)	4.277 (3.575)	2.444 (2.366)	2.679 (2.739)
Median	3	3	2	2

^a^Kuder-Richardson coefficient

For both populations, experienced stigma included items for being arrested, verbally harassed, blackmailed, physically abused, tortured, or forced to have sex. The most common perpetrators of forced sex among MSM were current or past male sexual partners or other MSM (data not shown). Police harassing the participant loaded on the experienced stigma factor for FSW.

Experienced healthcare stigma for both populations included being denied care, not treated well, or gossiped about by healthcare workers. Among MSM, difficulties accessing healthcare also loaded on the experienced healthcare stigma factor. Anticipated healthcare stigma included fear of or avoiding seeking healthcare. Stigma from family and friends included feeling excluded from family gatherings, gossiped about by family, or rejected by friends. Stigma from police among FSW included being refused police protection or experiencing, fearing, witnessing or hearing about police confiscation of condoms.

### Convergent validity

In both populations, higher stigma was generally significantly and positively associated with a family member or health worker knowing about involvement in sex work or same-sex practices, regardless of whether the disclosure was voluntary or involuntary (Table 8). The exception was among MSM in Lomé, who reported less stigma if they voluntarily disclosed to a health worker. Participants with greater cumulative reports of stigma had a greater likelihood of suicidal ideation, though this was not significant among MSM in Kara or FSW in Bobo-Dioulasso. FSW in Ouagadougou and both populations in Bobo-Dioulasso reported higher stigma if they participated in FSW, MSM or HIV-related organizations. Study participants in the MSM sample who identified as male had lower stigma scores than those who identified as female, transgender or intersex. The relationships between stigma and HIV testing and condom use were not consistent across cities and populations. Final items retained in each stigma metric are reported in Tables 9 and 10.

**Table 8:** Correlates of the FSW^a^ and MSM^b^ Stigma Metrics in Burkina Faso and Togo

	Ouagadougou		Bobo-Dioulasso		Lomé		Kara	
	OR	95% CI	OR	95% CI	OR	95% CI	OR	95% CI
FSW								
Told family about sex work	**1.12**	**1.03, 1.21**	0.97	0.90, 1.04	1.03	0.92, 1.15	**1.15**	**1.05, 1.26**
Family found out about sex work	**1.15**	**1.08, 1.24**	**1.44**	**1.31, 1.59**	**1.34**	**1.20, 1.50**	1.07	0.99, 1.16
Told health worker about sex work	**1.17**	**1.08, 1.27**	**1.09**	**1.02, 1.17**	0.98	0.90, 1.07	**1.15**	**1.06, 1.26**
Health worker found out about sex work	**1.12**	**1.02, 1.22**	1.10	0.99, 1.23	0.97	0.86, 1.10	**1.20**	**1.04, 1.39**
Ever had suicidal thoughts	**1.15**	**1.07, 1.24**	1.04	0.97, 1.11	**1.20**	**1.08, 1.33**	**1.26**	**1.14, 1.40**
Participated in FSW organization	**1.12**	**1.03, 1.21**	**1.30**	**1.20, 1.40**	0.97	0.87, 1.08	1.16	0.96, 1.40
Had condomless vaginal sex in the past 12 months	0.94	0.88, 1.00	**0.93**	**0.87, 0.99**	**1.15**	**1.04, 1.27**	1.06	0.98, 1.15
Ever tested for HIV more than once	1.04	0.97, 1.11	0.97	0.91, 1.03	1.07	0.98, 1.18	0.98	0.90, 1.06
MSM								
Told family about same-sex practices	**1.24**	**1.12, 1.37**	1.12	0.99, 1.25	**1.27**	**1.13, 1.43**	1.12	1.00, 1.26
Family found out about same-sex practices	**1.24**	**1.11, 1.38**	**1.35**	**1.19, 1.54**	**1.31**	**1.16, 1.48**	**1.35**	**1.19, 1.52**
Told health worker about same-sex practices	**1.27**	**1.14, 1.42**	**1.25**	**1.10, 1.41**	**0.87**	**0.77, 0.99**	**1.32**	**1.13, 1.54**
Health worker found out about same-sex practices	1.14	0.91, 1.42	1.18	0.91, 1.52	0.94	0.77, 1.14	**1.42**	**1.03, 1.96**
Ever had suicidal thoughts	**1.37**	**1.22, 1.55**	**1.45**	**1.26, 1.66**	**1.38**	**1.22, 1.58**	1.08	0.87, 1.33
Participated in MSM organization	1.05	0.94, 1.18	**1.25**	**1.07, 1.45**	1.02	0.92, 1.13	1.04	0.65, 1.67
Identifies as male	**0.84**	**0.76, 0.93**	**0.83**	**0.74, 0.93**	**0.75**	**0.66, 0.84**	**0.82**	**0.69, 0.96**
Ever had condomless anal sex	**1.10**	**1.00–1.20**	1.04	0.93, 1.17	**1.38**	**1.21, 1.59**	1.09	0.98, 1.23
Ever tested for HIV more than once	1.04	0.96, 1.14	**1.16**	**1.04, 1.30**	1.06	0.95, 1.18	0.95	0.85, 1.06

^a^female sex worker

^b^men who have sex with men; bold text indicates significance at the *p*<0.05 level

**Table 9:** Final items included in the female sex worker stigma metric

1. Have police ever harassed or intimidated you for being a sex worker?	
2. Were you ever arrested on charges related to sex work?	
3. Have you ever been verbally harassed and felt it was because you sell sex?	
4. Have you ever been blackmailed by someone because you sell sex?	
5. Have you ever been physically aggressed (pushed, shoved; slapped; hit; kicked; choked; or otherwise physically hurt)? Do you believe any of these experience(s) of physical violence was/were related to the fact that you sell sex?	
6. Have you ever been tortured by someone? If yes, do you believe this was because you sell sex?	
7. Have you ever been forced to have sex when you did not want to? (By forced, I mean physically forced, coerced to have sex, or penetrated with an object, when you did not want to). Do you believe any of these experiences of sexual violence were related to the fact that you sell sex?	
8. Have you ever been denied health services (or someone kept you from receiving health services) because you sell sex?	
9. Have you ever felt that you were not treated well in a health center because you sell sex?	
10. Have you ever heard healthcare providers gossiping about you because you sell sex?	
11. Have you ever felt afraid to go to healthcare services because you worry someone may learn you sell sex?	
12. Have you ever avoided going to healthcare services because you worry someone may learn you sell sex?	
13. Have you ever felt excluded from family gatherings because you sell sex?	
14. Have you ever felt that family members have made discriminatory remarks or gossiped about you because you sell sex?	
15. Have you ever felt rejected by your friends because you sell sex?	
16. Have you ever felt that the police refused to protect you because you sell sex?	
17. Have you ever avoided carrying condoms because you were afraid that they might get you in trouble with the police?	
18. Has a police officer ever taken condoms away from you, thrown them on the ground or in the garbage?	
19. Have you ever witnessed (i.e. seen) police confiscating or destroying condoms held by a sex worker or outreach worker?	
20. Have you ever heard about incidents when police confiscated or destroyed condoms held by other sex workers or by outreach workers?

**Table 10:** Final items included in the men who have sex with men stigma metric

1. Were you ever arrested on charges of homosexuality [or other related charge]?	
2. Have you ever been verbally harassed and felt it was because you have sex with men?	
3. Have you ever been blackmailed by someone because you have sex with men?	
4. Have you ever been physically aggressed (pushed, shoved; slapped; hit; kicked; choked; or otherwise physically hurt)? Do you believe any of these experience(s) of physical violence was/were related to the fact that you have sex with men?	
5. Have you ever been tortured by someone? If yes, do you believe this was because you have sex with men?	
6. Have you ever been forced to have sex when you did not want to? (By forced, I mean physically forced, coerced to have sex, or penetrated with an object, when you did not want to). Do you believe any of these experiences of sexual violence were related to the fact that you have sex with men?	
7. Have you ever been denied health services (or someone kept you from receiving health services) because you have sex with men?	
8. Have you ever felt that you were not treated well in a health center because someone knew that you have sex with men?	
9. Have you ever heard healthcare providers gossiping about you because you have sex with men?	
10. Have you ever had difficulties in accessing healthcare services because you have sex with men?	
11. Have you ever felt afraid to go to healthcare services because you worry someone may learn you have sex with men?	
12. Have you ever avoided going to healthcare services because you worry someone may learn you have sex with men?	
13. Have you ever felt excluded from family gatherings because you have sex with men?	
14. Have you ever felt that family members have made discriminatory remarks or gossiped about you because you have sex with men?	
15. Have you ever felt rejected by your friends because you have sex with men?	
16. Have you ever felt that the police refused to protect you because you have sex with men?	

## Discussion

The results of this multi-country study illustrate the potential for brief tools to measure stigma among key populations that work well across country contexts and languages. These measures include items related to perceived, anticipated and enacted types of stigma. The sources of stigma represent multiple levels of the social ecological model of HIV risk among key populations, including social (family and friends), community (healthcare workers), and policy (police) [14]. Though many factors were common to both populations, additional questions asked of FSW about interactions with police constituted a separate factor relevant to the overall construct of stigma within this population. The metrics were significantly related to disclosure of sex work or same-sex practices to family or a health worker, suicidal ideation, organization participation, and gender identity.

These analyses build on earlier studies using some of the same questionnaire items. Previous studies have used these indicators individually in models as independent variables [75, 109], or reported dichotomous aggregate stigma measures such as any social stigma [73, 75, 79]. Dichotomized measures may not fully capture the granularity of dose-response relationships between the number of stigma events reported and health outcomes.

In this study experienced stigma included both physically violent and emotionally abusive experiences, demonstrating a wide range of severity for this factor. Sexual violence related to being MSM loaded on the same factor as blackmail. Perpetrators (including other MSM) may believe they will not be prosecuted because the police will not take action, or the person will not report the rape due to being MSM.

Among FSW, being arrested for selling sex loaded on the experienced stigma factor rather than the stigma from police factor. In countries where aspects of sex work are criminalized, arrests may indicate structural stigma from the government or society rather than from police doing their job. The items that loaded on the stigma from police factor included some that could be categorized as anticipated stigma (Have you ever avoided carrying condoms because you were afraid that they might get you in trouble with the police?), some that could be categorized as perceived stigma (Have you ever felt that the police refused to protect you because you sell sex?), and some that could be categorized as enacted stigma (police confiscation of condoms). Studies in the United States and human rights reports have provided some indications of police confiscation of condoms as evidence of sex work [110–112]. This study presented quantitative data from a diverse sample of FSW on the prevalence of experiencing, witnessing, hearing about, and avoiding carrying condoms due to police confiscation. Given the emergence of stigma from police as one components of stigma in the metric for FSW specifically, using research and evaluation to identify effective approaches in these countries’ contexts to prevent harmful policing practices through education, policy, or increased accountability appears to be warranted [113, 114].

The stigma metrics showed promising convergent validity in that in bivariate models they generally performed as had been theorized based on conceptual models and existing literature. The findings that higher stigma scores were positively associated with a family member or health worker knowing about participants’ involvement in sex work or same-sex practices is consistent with prior studies in The Gambia and the United States [75, 105]. Similarly, stigma was positively associated with suicidal ideation, as other researchers have reported in China [32] and the United States [106]. Transgender participants accrued into the studies focused on MSM reported higher stigma than cisgender MSM, which may be due to intersecting stigma associated with sexual practices and gender identity. MSM who are more masculine or hide their sexual orientation or practices may even reject those who are more open about their sexual orientation or practices or considered more feminine. Given the unique needs of transgender and cisgender populations, valid and reliable stigma measures are needed specifically for transgender women [110, 111].

This study has several limitations. Findings may not be generalizable to settings outside the cities in West Africa included in this study. This is a secondary analysis of data collected for a study measuring HIV prevalence among key populations. For MSM, the inclusion criteria of having anal sex with a man in the past year was chosen due to its relationship with potential HIV acquisition or transmission risk. Items in this stigma metric may not capture stigma targeted toward MSM communities or identities rather than sexual practices. Individuals who did not meet the inclusion criteria but are in same-sex relationships or identify as sexually diverse, sexually attracted to the same gender, gay or bisexual may face similar or different types of stigma compared to those included in this study.

All self-reported variables may be subject to social desirability bias and inaccurate recall. In the context of limited disclosure of sex work or same-sex practices, stigma may be lower than expected because participants’ families, friends, healthcare providers or others are not aware of these practices. However, MSM may also experience stigma because of their relationships, gender non-conformity, mannerisms, or friends who are also sexual minorities. For example, others may know that the participant is not dating a woman, getting married, acting “masculine”, or fulfilling other heteronormative expectations, and this may be another source of stigma. This study investigated stigma from the perspective of MSM and FSW, which may differ from stigma toward these groups reported by others. Future research measuring stigma from both perspectives of those within and outside the population of interest may inform stigma reduction programs. If, for instance, MSM or FSW report experiencing stigma from healthcare workers but healthcare providers do not report stigmatizing attitudes, making these providers aware of actions that may be unintentionally stigmatizing could result in improved services.

Because of the question wording, it is not possible to determine whether issues related to stigma from family and friends were experienced (e.g., the participant actually heard the discriminatory remarks) or perceived (e.g., the participant heard a family member whispering to another and assumed it was about the participant selling sex). Other items potentially assessing felt normative or perceived stigma (e.g., Have you ever felt scared to walk around in public places because you sell sex?) were excluded for the reasons mentioned above. Some studies have shown that perceived stigma is more commonly reported than other types of stigma [115] and is significantly and negatively associated with quality of life [116]. Questionnaire items excluded from these metrics may be important stigma indicators that should be assessed in studies in different contexts. These metrics do not specifically measure secondary stigma, with mental health being measured in lieu of internalized stigma. Internalized stigma is often preceded by experienced stigma [117] and has been shown to be less closely correlated with avoiding or delaying seeking healthcare than experienced stigma [17]. Future analyses could leverage these developed metrics to explore intersectionality through intra- and inter-categorical approaches.

Potential confounding variables were not included in these analyses because the primary purpose was the development of the metrics. Stratified analyses by age, HIV status, and socioeconomic status will enable comparisons to better understand the effects of privilege and marginalization and the distribution of the burden of stigma [113]. Future studies should use confirmatory factor analysis to assess whether the metrics’ structure is applicable in other samples of key populations across SSA. Moreover, additional approaches for validating and assessing the metrics’ psychometric properties including assessing predictive validity and test-retest reliability would add strength to the conclusions.

## Conclusions

There are ongoing and planned stigma mitigation interventions in SSA that would benefit by the ability to consistently measure stigma across key populations and over time using these reliable and valid stigma measures [22, 114]. Stigma may be addressed through implementing nondiscrimination policies and increasing police protection of vulnerable populations to hold perpetrators of violence and blackmail accountable. Comprehensive stigma reduction interventions should also work directly with MSM and FSW individuals and community groups to address stigma, given the level of anticipated stigma found in this study and association between stigma and suicidal ideation. Continued stigma measurement and evaluation of stigma reduction interventions must urgently be addressed in order to realize the goals of achieving an AIDS-Free generation in our lifetimes.

## Abbreviations

AIDS: Acquired Immune Deficiency Syndrome
FSW: Female sex workers
HIV: Human Immunodeficiency Virus
MSM: Men who have sex with men
SSA: Sub-Saharan Africa
WEIRD: Western, Educated, Industrialized, Rich, or Democratic
